# Reflective practice and compassion fatigue among hemodialysis specialist nurses in Inner Mongolia: a cross-sectional study

**DOI:** 10.3389/fpsyg.2026.1919032

**Published:** 2026-09-21

**Authors:** Bing Wen, Ya Tuo, Yi Li

**Affiliations:** 1 Department of Hemodialysis, Hohhot Hospital of Traditional Chinese Medicine and Mongolian Medicine, Hohhot, Inner Mongolia, China; 2 Department of Nursing, The First Hospital of Hohhot, Hohhot, Inner Mongolia, China

**Keywords:** compassion fatigue, cross-sectional studies, hemodialysis, nurses, reflective practice

## Abstract

**Objective:**

To describe reflective practice and compassion fatigue among hemodialysis specialist nurses in Inner Mongolia and examine their association.

**Methods:**

We conducted a cross-sectional online survey from August to October 2025 using convenience sampling. Participants completed a demographic questionnaire, the Chinese Reflective Practice Questionnaire, and the 36-item Compassion Fatigue Scale for Healthcare Workers. Descriptive statistics, Mann–Whitney U tests, Kruskal–Wallis tests, Spearman correlations, and exploratory multiple linear regression were used.

**Results:**

Among 249 participants, the mean reflective practice score was 148.29 ± 27.81 and the mean compassion fatigue score was 72.39 ± 22.29 (mean item score, 2.01 ± 0.62). Reflective practice was inversely correlated with compassion fatigue (*r* = −0.436, *p* < 0.01). In the regression model, associate chief nurses had lower reflective practice scores than nurses (*B* = −24.405, 95% CI − 42.105 to −6.706, *p* = 0.007), and higher compassion fatigue scores were associated with lower reflective practice scores (*B* = −0.586, 95% CI − 0.727 to −0.444, *p* < 0.001). The overall model fit was R^2^ = 0.237, adjusted R^2^ = 0.200, *F*(11, 237) = 6.692, *p* < 0.001.

**Conclusion:**

Reflective practice and compassion fatigue were inversely associated in this sample. Longitudinal and intervention studies are needed before temporal, causal, or intervention effects can be inferred.

## 1 Introduction

Hemodialysis nursing involves sustained technical demands, repeated patient contact, and rapid clinical judgment. Compassion fatigue has been documented among hemodialysis nurses in China and has been associated with work engagement and turnover intention (Wang et al., 2022; Cao and Chen, 2021).

Reflective practice is the deliberate examination of clinical experience to inform subsequent judgment and action (Patel and Metersky, 2022; Priddis and Rogers, 2018). It may help nurses process demanding clinical encounters, although unsupported or self-critical reflection may also add to perceived strain. Reflective practice and compassion fatigue are therefore conceptually related, but a cross-sectional study cannot establish their temporal order or causal direction.

Research has examined reflective practice among Chinese specialist nurses (Xu et al., 2024), but evidence focused on certified hemodialysis specialist nurses in Inner Mongolia remains limited. This study therefore described reflective practice and compassion fatigue among hemodialysis specialist nurses across 12 cities and examined their association. We asked: (1) What are the levels of reflective practice and compassion fatigue in this population? (2) Are the two constructs associated? (3) Which measured demographic and occupational characteristics are associated with reflective practice in an exploratory multivariable model?

## 2 Subjects and methods

### 2.1 Study participants

We conducted a cross-sectional study of hemodialysis specialist nurses (HDSNs) working in units across 12 cities in the Inner Mongolia Autonomous Region: Hohhot, Baotou, Hulun Buir, Xing’an, Tongliao, Chifeng, Xilingol, Ulanqab, Ordos, Bayannur, Wuhai, and Alxa. Data were collected from August 14 to October 25, 2025. Convenience sampling was used because no complete regional sampling frame of certified HDSNs was available and recruitment had to be coordinated through participating dialysis units. This approach allowed broad geographic coverage but may have introduced selection bias and limits generalizability.

Eligible participants (1) held a certificate confirming completion of a hemodialysis specialist training program approved by the Chinese Nursing Association or the Inner Mongolia Autonomous Region Nursing Association, (2) had at least 1 year of clinical experience in hemodialysis nursing, and (3) provided electronic informed consent. Interns, trainees, and nurses who were on leave or rotating outside their unit during the study period were excluded.

The Ethics Committee of the First Hospital of Hohhot, Inner Mongolia, approved the study (IRB2024289). The first survey page described the study, and participants provided electronic informed consent before proceeding. Of 255 submitted questionnaires, 249 were included in the analysis (valid-response rate, 97.6%). Six questionnaires were excluded under the prespecified quality controls described in Section 2.3.

### 2.2 Measures

#### 2.2.1 Demographic questionnaire

A brief self-administered questionnaire collected age, sex, educational attainment, professional title, marital status, number of children, years of dialysis-center experience, overtime frequency, bed-to-nurse ratio, and employment type.

#### 2.2.2 Reflective Practice Questionnaire (RPQ)

The original 40-item RPQ was developed by Priddis and Rogers to assess reflective practice and related constructs across professional settings (Priddis and Rogers, 2018). We used the 33-item Chinese version cross-culturally adapted and validated among specialist nurses by Wu et al. (2022). It contains five domains: Reflective Practice Ability and Improvement Drive (14 items), Confidence (7 items), Job Satisfaction (4 items), Reflective Practice Acuity (5 items), and Stress Induced by Reflective Practice (3 items). Items are rated from 1 (strongly disagree) to 6 (strongly agree), giving a total score range of 33–198. Higher total scores indicate greater reflective practice. Cronbach’s α was 0.950 in the Chinese validation study and 0.969 in the present sample.

#### 2.2.3 Compassion fatigue scale for healthcare workers

We used the Compassion Fatigue Scale for Healthcare Workers developed by Li for Chinese healthcare personnel (Li, 2011). The instrument contains 36 items across six domains: Mental Tension, Loss of Enthusiasm, Negative Behavior, Emotional Apathy, Self-doubt of Ability, and Loss of Fighting Spirit. Each item is rated from 1 (strongly disagree) to 5 (strongly agree), giving a total score range of 36–180. Higher total scores indicate greater compassion fatigue. Cronbach’s α was 0.858 in the original study and 0.951 in the present sample. To maintain a single scoring convention throughout this manuscript, all reported compassion fatigue totals and item means are based on this 36-item version.

### 2.3 Data collection

The survey was administered through Questionnaire Star. Investigators first contacted the heads of participating hemodialysis departments, who distributed a survey link or QR code to eligible nurses through existing WeChat groups. Participation was voluntary and anonymous. The first page stated the study purpose, eligibility criteria, instructions, and consent information. All items required a response. The platform restricted submissions to one per IP address, and responses completed in less than 5 min were excluded. The research team also screened submissions for eligibility and completeness. These controls reduced but could not eliminate duplicate participation, volunteer bias, or inaccurate self-report.

### 2.4 Statistical analysis

Analyses were performed in SPSS version 26.0. Distributional form was assessed with the Shapiro–Wilk test. Continuous variables are reported as mean ± standard deviation and, where appropriate, median and interquartile range. Two-group comparisons used the Mann–Whitney U test, and comparisons across three or more groups used the Kruskal–Wallis H test. Associations between reflective practice and compassion fatigue were assessed with Spearman’s rank correlation. Because Table 1 involved multiple exploratory subgroup comparisons, these *p* values were interpreted as hypothesis-generating; a Bonferroni threshold of 0.0033 (0.05/15 comparisons per outcome) was used as a sensitivity criterion.

**Table 1:** Comparison of reflective practice scores across demographic and professional characteristics (*n* = 249).

Characteristic	Category	n	Reflective practice			Compassion fatigue score		
			Score ( $\bar{x}\pm s$ )	Test statistic	*P*	Score ( $\bar{x}\pm s$ )	Test statistic	*P*
Gender	Male	35	153.23 ± 27.64 148 (133, 179)	-1.100b	0.271	72.37 ± 24.04 74 (49, 88)	−0.028b	0.978
	Female	214	147.49 ± 27.82 146 (128, 168)			72.39 ± 22.04 73 (53, 86)		
Age group	26 ~ 30 years	27	147.63 ± 28.02 144 (129, 171)	0.158a	0.984	73.22 ± 28.69 74 (44, 97)	2.144a	0.543
	31 ~ 40 years	154	148.86 ± 27.72 146 (130, 168)			73.58 ± 22.01 74 (56, 86)		
	41 ~ 50 years	60	147.97 ± 27.27 148.5 (128, 170)			68.78 ± 20.32 69.5 (52, 83.75)		
	51 ~ 60 years	8	142 ± 37.09 149.5 (107, 173)			73.50 ± 17.76 80.5 (55, 86)		
Education level	Secondary school or below	3	132.33 ± 38.19 124 (99, 174)	1.004b	0.315	67.33 ± 21.78	0.311b	0.756
	College degree or above	246	148.49 ± 27.71 147 (129, 169)			72.45 ± 22.32 73 (53, 86)		
Marital status	Married	221	148.99 ± 27.63 147 (130, 169)	1.783a	0.410	71.89 ± 21.86 73 (53, 86)	1.893a	0.388
	Unmarried	21	144.52 ± 28.71 137 (129, 160)			79.76 ± 27.46 77 (54.5, 102.5)		
	Divorced	7	137.71 ± 32.1 129 (114, 176)			65.86 ± 15.55 73 (48, 74)		
Children	None	35	144.51 ± 25.56 140 (129, 161)	0.723a	0.868	77.63 ± 25.98 77 (55, 100)	3.464a	0.325
	One	155	148.96 ± 28.95 147 (128, 171)			72.44 ± 22.06 74 (56, 86)		
	Two	58	148.72 ± 26.47 145.5 (129, 171)			68.76 ± 20.16 72 (50, 86)		
	Three	1	152					
Years in dialysis center	≤5 years	52	148.15 ± 31.28 148 (129.25, 170.25)	2.554a	0.466	70.33 ± 24.68 65 (48.5, 91.5)	11.357a	0.010
	6–10 years	69	150.88 ± 26.47 154 (134, 171)			69.30 ± 22.09 72 (49.5, 85.5)		
	11–15 years	89	145.94 ± 26.02 142 (127, 163)			78.65 ± 21.66 81 (64, 89)		
	16–20 years	39	149.26 ± 29.79 150 (129, 172)			66.28 ± 17.59 70 (50, 82)		
Professional title	Nurse	13	151.62 ± 34.72 163 (128.5, 177)	3.935a	0.415	80.46 ± 31.62 77 (49, 111.5)	2.277a	0.685
	Senior nurse	57	152.19 ± 31.14 152 (131, 177)			75.61 ± 25.38 75 (49.5, 94.5)		
	Charge nurse	129	148.35 ± 25.94 145 (130, 165)			70.92 ± 20.96 70 (53, 85.5)		
	Associate chief nurse	45	142 ± 27.63 142 (117, 167.5)			70.60 ± 18.69 77 (54, 85.5)		
	Chief nurse	5	150.4 ± 11.61 149 (142, 159.5)			68.40 ± 19.40 76 (49.5, 83.5)		
Overtime frequency (times/week)	0	112	148.99 ± 28.62 147 (129, 171)	1.509a	0.680	70.84 ± 22.72 70 (50.5, 86)	2.740a	0.434
	1–2	111	146.51 ± 26.35 145 (128, 165)			73.94 ± 21.90 76 (56, 87)		
	3–4	23	152.74 ± 31.99 157 (127, 182)			70.65 ± 22.66 74 (49, 86)		
	≥5	3	154 ± 22.91			86.00 ± 17.35		
Employment type	Contract	174	148.58 ± 27.34 146.5 (130, 169)	0.107a	0.948	72.87 ± 21.97 73 (55, 86)	0.534a	0.766
	Contract (special)	19	148.68 ± 27.48 136 (127, 179)			69.11 ± 24.93 73 (43, 84)		
	Permanent	56	147.27 ± 29.8 149 (124.25, 169)			71.98 ± 22.64 74.5 (52, 86)		

Data are presented as mean ± SD and median (IQR), where available. Superscript a indicates the Kruskal–Wallis H test; superscript b indicates the Mann–Whitney U test. Because 15 subgroup comparisons were conducted for each outcome, *p* < 0.0033 was used as a Bonferroni sensitivity threshold. SD, standard deviation; IQR, interquartile range.

An exploratory multiple linear regression used total reflective practice score as the dependent variable. Candidate variables were selected *a priori* for demographic or occupational relevance and included bed-to-nurse ratio, years of dialysis-center experience, educational attainment, professional title, overtime frequency, employment type, and total compassion fatigue score. Categorical predictors were entered using indicator coding, with Nurse as the reference for professional title and Contract as the reference for employment type. Tolerance and variance inflation factor (VIF) were examined for multicollinearity. Regression assumptions were evaluated using residual-versus-predicted plots, a normal probability plot, the Shapiro–Wilk test of residuals, the Durbin–Watson statistic, standardized residuals, leverage, and Cook’s distance. All tests were two-sided. *p* < 0.05 was considered statistically significant, except for the multiple-comparison sensitivity criterion noted above.

No *a priori* sample-size calculation was performed because all eligible respondents available through participating units during the study period were invited. A sensitivity calculation based on *n* = 249, two-sided *α* = 0.05, and 80% power indicated that the sample could detect a correlation of approximately |r| = 0.18; this calculation does not replace an *a priori* design calculation.

## 3 Results

### 3.1 Descriptive statistics of reflective practice and compassion fatigue

For the 249 HDSNs in our sample, the total RPQ had a mean of 148.29 ± 27.81, with a mean item score of 4.49 ± 0.84. The 36-item compassion fatigue total had a mean of 72.39 ± 22.29, corresponding to a mean item score of 2.01 ± 0.62. Table 2 presents the internally consistent total scores. Dimension-level compassion fatigue values from the earlier draft were removed because they did not correspond to the stated 36-item scoring structure.

**Table 2:** Reflective practice and compassion fatigue scores among hemodialysis specialist nurses (*n* = 249).

Domain	Score (mean ± SD)	Item mean score (mean ± SD)
Reflective practice – total score	148.29 ± 27.81	4.49 ± 0.84
Reflective practice ability and improvement drive	65.55 ± 12.83	4.68 ± 0.92
Confidence	31.65 ± 6.91	4.52 ± 0.99
Job satisfaction	17.96 ± 4.3	4.49 ± 1.07
Reflective practice acuity	23.11 ± 4.39	4.62 ± 0.88
Stress induced by reflective practice	10.01 ± 3.83	3.34 ± 1.28
Compassion fatigue – total score	72.39 ± 22.29	2.01 ± 0.62

### 3.2 Bivariate analysis across demographic subgroups

Shapiro–Wilk tests indicated departures from normality in subgroup distributions (all *p* < 0.05). We therefore used nonparametric tests for between-group comparisons. Table 1 reports both mean ± SD and median (IQR). Under the Bonferroni sensitivity threshold (*p* < 0.0033), none of the subgroup comparisons met the corrected criterion.

### 3.3 Correlation between reflective practice and compassion fatigue

Total reflective practice was inversely associated with total compassion fatigue (*r* = −0.436, *p* < 0.01) and with Mental Tension (*r* = −0.314), Loss of Enthusiasm (*r* = −0.433), Negative Behavior (*r* = −0.388), Emotional Apathy (*r* = −0.401), Self-doubt of Ability (*r* = −0.389), and Loss of Fighting Spirit (*r* = −0.392; all *p* < 0.01). Table 3 presents the full correlation matrix.

**Table 3:** Spearman correlations between reflective practice and compassion fatigue among hemodialysis specialist nurses.

Domain	Compassion fatigue – total score	Mental tension	Loss of enthusiasm	Negative behavior	Emotional apathy	Self-doubt of ability	Loss of fighting spirit
Reflective practice – total score	−0.436^**^	−0.314^**^	−0.433^**^	−0.388^**^	−0.401^**^	−0.389^**^	−0.392^**^
Reflective practice ability and improvement drive	−0.474^**^	−0.332^**^	−0.475^**^	−0.433^**^	−0.440^**^	−0.421^**^	−0.416^**^
Confidence	−0.423^**^	−0.275^**^	−0.431^**^	−0.401^**^	−0.407^**^	−0.376^**^	−0.364^**^
Job satisfaction	−0.523^**^	−0.422^**^	−0.431^**^	−0.427^**^	−0.446^**^	−0.493^**^	−0.542^**^
Reflective practice acuity	−0.387^**^	−0.294^**^	−0.402^**^	−0.328^**^	−0.351^**^	−0.348^**^	−0.330^**^
Stress induced by reflective practice	0.211^**^	0.137^*^	0.168^**^	0.213^**^	0.199^**^	0.216^**^	0.189^**^

*p < 0.05; **p < 0.01. Values are Spearman correlation coefficients.

### 3.4 Factors associated with reflective practice: exploratory multiple linear regression

The exploratory model included bed-to-nurse ratio, years of dialysis-center experience, educational attainment, professional title, overtime frequency, employment type, and total compassion fatigue score. Total reflective practice score was the outcome. Table 4 shows the coding of categorical variables, and Table 5 reports the coefficient estimates. Compared with nurses, associate chief nurses had lower reflective practice scores (*B* = −24.405, 95% CI − 42.105 to −6.706, *p* = 0.007). Higher compassion fatigue scores were also associated with lower reflective practice scores (*B* = −0.586, 95% CI − 0.727 to −0.444, *p* < 0.001). These estimates describe adjusted associations, not causal effects. The model explained 23.7% of the variance in reflective practice scores: R^2^ = 0.237, adjusted R^2^ = 0.200, *F*(11, 237) = 6.692, *p* < 0.001 (Table 6). Residual diagnostics were Durbin–Watson = 1.89, Shapiro–Wilk W = 0.992 (*p* = 0.214), maximum Cook’s distance = 0.083, and maximum leverage = 0.071. Inspection of residual-versus-predicted and normal probability plots indicated no material violation of linearity, homoscedasticity, or residual normality.

**Table 4:** Assignment of independent variables in the multivariate analysis of factors influencing reflective practice among hemodialysis specialist nurses.

Independent variable	Assignment
Bed-to-nurse ratio	1 = 1:3; 2 = 1:4; 3 = 1:5; 4 = 1:6; 5 = 1:7
Years of experience in dialysis center	1 = 1–5 years; 2 = 6–10 years; 3 = 11–15 years; 4 = 16–20 years
Education level	1 = Secondary school or below; 2 = College degree or above
Professional title	1 = Nurse; 2 = Senior nurse; 3 = Charge nurse; 4 = Associate chief nurse; 5 = Chief nurse
Overtime frequency	1 = 0 times/week; 2 = 1–2 times/week; 3 = 3–4 times/week; 4 = ≥5 times/week
Employment type	1 = Contract; 2 = Contract (special); 3 = Permanent

**Table 5:** Exploratory multiple linear regression of reflective practice scores (*n* = 249).

Variable	B	SE	Std. β	t	*P*	95% CI	Tolerance	VIF
Constant	143.497	29.767	—	4.821	<0.001	84.856 to 202.138	—	—
Bed-to-nurse ratio	1.599	1.541	0.064	1.038	0.300	−1.436 to 4.635	0.893	1.120
Years in dialysis center	2.603	1.932	0.091	1.347	0.179	−1.203 to 6.409	0.674	1.483
Education level	23.833	14.828	0.071	1.607	0.109	−5.378 to 53.044	0.942	1.061
Professional title (ref: Nurse)								
Senior nurse	−5.757	7.818	−0.089	−0.736	0.462	−21.157 to 9.644	0.229	4.374
Charge nurse	−13.844	7.619	−0.213	−1.817	0.070	−28.854 to 1.166	0.170	5.878
Associate chief nurse	−24.405	8.985	−0.338	−2.716	0.007	−42.105 to −6.706	0.206	4.847
Chief nurse	−17.758	13.869	−0.079	−1.280	0.202	−45.081 to 9.565	0.651	1.535
Overtime frequency	1.023	2.365	0.026	0.433	0.666	−3.636 to 5.681	0.920	1.087
Employment type (ref: Contract)								
Contract (special)	−2.475	6.068	−0.023	−0.408	0.684	−14.430 to 9.480	0.950	1.053
Permanent	3.408	4.177	0.051	0.816	0.415	−4.821 to 11.636	0.811	1.233
Compassion fatigue score	−0.586	0.072	−0.336	−8.155	<0.001	−0.727 to −0.444	0.967	1.035

R^2^ = 0.237; adjusted R^2^ = 0.200; F(11, 237) = 6.692, p < 0.001; Durbin–Watson = 1.89. Maximum Cook’s distance = 0.083; maximum leverage = 0.071.

**Table 6:** ANOVA for the exploratory multiple linear regression.

Source	Sum of squares	df	Mean square	*F*	*P*
Regression	45,457.129	11	4,132.466	6.692	<0.001
Residual	146,345.104	237	617.490		
Total	191,802.233	248			

Regression equation: Predicted reflective practice score = 143.497 + 1.599(bed-to-nurse ratio) + 2.603(years of dialysis-center experience) + 23.833(education level) − 5.757(senior nurse) − 13.844(charge nurse) − 24.405(associate chief nurse) − 17.758(chief nurse) + 1.023(overtime frequency) − 2.475(contract-special) + 3.408(permanent employment) − 0.586(total compassion fatigue score).

## 4 Discussion

### 4.1 Reflective practice among hemodialysis specialist nurses

The mean reflective practice score was 148.29 ± 27.81 (item mean, 4.49 ± 0.84). This value was lower than the 158.60 ± 26.10 reported in a recent study of specialist nurses from six tertiary hospitals in Xiamen (Xu et al., 2024). Differences in specialty mix, organizational context, and sampling may contribute to this contrast, but the present study did not directly test those explanations. Reflective Practice Ability and Improvement Drive and Reflective Practice Acuity were among the higher-scoring domains. Structured reflection, protected time, mentorship, and psychologically safe discussion are reasonable approaches for future evaluation, but their effects cannot be inferred from this cross-sectional survey (Patel and Metersky, 2022).

### 4.2 Compassion fatigue among hemodialysis specialist nurses

The mean compassion fatigue score was 72.39 ± 22.29 (item mean, 2.01 ± 0.62) on the 36-item scale. Previous Chinese studies of hemodialysis nurses have used different compassion-fatigue instruments and scoring systems (Wang et al., 2022; Cao and Chen, 2021), so direct numerical comparisons would be misleading. The present findings should therefore be interpreted within the scoring framework described in Section 2.2.3. Contextual factors may contribute to differences across settings, but these factors were not measured in this survey.

The observed compassion fatigue level underscores the importance of evaluating occupational support in hemodialysis settings. However, this study did not test staffing changes, psychological support, or reflective-practice training. The effectiveness of these approaches should be evaluated in prospective intervention studies rather than inferred from the present associations.

### 4.3 Professional title and reflective practice

In the exploratory regression, associate chief nurses had lower reflective practice scores than nurses after adjustment for the measured covariates (*B* = −24.405, 95% CI − 42.105 to −6.706, *p* = 0.007). Other professional-title categories did not differ statistically from the reference group. This finding does not show that seniority reduces reflective practice. Unmeasured role responsibilities, workload, or organizational factors may contribute, and the small numbers in several title categories reduce estimate stability. Future studies should measure these factors directly before title-specific interventions are considered.

### 4.4 Association between reflective practice and compassion fatigue

Reflective practice and compassion fatigue were inversely correlated (*r* = −0.436, *p* < 0.01). Job Satisfaction showed the strongest inverse correlation with total compassion fatigue (*r* = −0.523, *p* < 0.01). These results indicate that the constructs varied together within this sample. They do not establish that reflective practice reduces compassion fatigue or that compassion fatigue depletes reflective practice. Shared workplace conditions, personal characteristics, and common method variance may also contribute to the association.

Stress Induced by Reflective Practice was positively correlated with compassion fatigue (*r* = 0.211, *p* < 0.01). Unsupported or self-critical reflection may add to perceived burden, while nurses experiencing greater compassion fatigue may also perceive reflection as more stressful. Because both explanations are compatible with the cross-sectional data, temporal direction cannot be determined. Longitudinal studies with repeated measurements are needed to distinguish these pathways.

## 5 Conclusion and limitations

Among 249 hemodialysis specialist nurses from 12 cities in Inner Mongolia, reflective practice and compassion fatigue were inversely associated. In an exploratory adjusted model, associate chief nurse status and higher compassion fatigue scores were associated with lower reflective practice scores. These findings identify relationships for further study, but they do not show that compassion fatigue causes lower reflective practice or that reflective-practice training reduces compassion fatigue.

Several limitations should be considered. The cross-sectional design prevents temporal or causal inference and leaves open the possibility of reverse causality. Convenience sampling and voluntary online participation may have introduced selection and volunteer bias. Nurses who were more engaged with professional networks or more willing to discuss workplace experiences may have been more likely to participate. The sample was drawn from one autonomous region, so the findings should not be generalized to hemodialysis nurses in other Chinese regions or countries without further study. Both primary variables were self-reported in the same questionnaire, creating risks of recall, social-desirability, information, and common method bias. Small numbers in some cities and professional-title categories reduce the stability of subgroup estimates. No *a priori* sample-size calculation was performed, and the multivariable findings should be treated as exploratory.

Future research should use multicenter probability samples, prospective designs, and repeated measurements to clarify temporal pathways between reflective practice and compassion fatigue. Qualitative studies could examine how local professional norms, organizational conditions, and culturally specific values shape nurses’ experiences. Those findings could then inform culturally responsive reflective-practice or support interventions, which should be tested before implementation effects are claimed.

## Data Availability

The original contributions presented in the study are included in the article/supplementary material, further inquiries can be directed to the corresponding author.
